# The impact of topical lidocaine and timing of LMA removal on the incidence of airway events during the recovery period in children: a randomized controlled trial

**DOI:** 10.1186/s12871-021-01235-7

**Published:** 2021-01-08

**Authors:** Ruiqiang Sun, Xiaoyun Bao, Xuesong Gao, Tong Li, Quan Wang, Yueping Li

**Affiliations:** 1 Department of Anesthesiology, Tianjin Eye Hospital, No. 4 Gansu Road, Heping District, Tianjin, 300022 China; 2 Tianjin Huaming Community Healthcare Service Center, Tianjin, China

**Keywords:** Laryngeal masks, Lidocaine, Child, General anesthesia, Airway events

## Abstract

**Background:**

The timing of laryngeal mask airway (LMA) removal remains undefined. This study aimed to assess the optimal timing for LMA removal and whether topical anesthesia with lidocaine could reduce airway adverse events.

**Methods:**

This randomized controlled trial assessed one-to-six-year-old children with ASA I-II scheduled for squint correction surgery under general anesthesia. The children were randomized into the LA (lidocaine cream smeared to the cuff of the LMA before insertion, with mask removal in the awake state), LD (lidocaine application and LMA removal under deep anesthesia), NLA (hydrosoluble lubricant application and LMA removal in the awake state) and NLD (hydrosoluble lubricant application and LMA removal in deep anesthesia) groups. The primary endpoint was a composite of irritating cough, laryngeal spasm, SpO_2_ < 96%, and glossocoma in the recovery period in the PACU. The secondary endpoints included the incidence of pharyngalgia and hoarseness within 24 h after the operation, duration of PACU stay, and incidence of agitation in the recovery period. The assessor was unblinded.

**Results:**

Each group included 98 children. The overall incidence of adverse airway events was significantly lower in the LA group (9.4%) compared with the LD (23.7%), NLA (32.6%), and NLD (28.7%) groups (*P*=0.001). Cough and laryngeal spasm rates were significantly higher in the NLA group (20.0 and 9.5%, respectively) than the LA (5.2 and 0%, respectively), LD (4.1 and 1.0%, respectively), and NLD (9.6 and 2.1%, respectively) groups (*P*=0.001). Glossocoma incidence was significantly lower in the LA and NLA groups (0%) than in the LD (19.6%) and NLD (20.2%) groups (*P*< 0.001). At 24 h post-operation, pharyngalgia incidence was significantly higher in the NLA group (15.8%) than the LA (3.1%), LD (1.0%), and NLD (3.2%) groups (*P*< 0.001).

**Conclusions:**

LMA removal in the awake state after topical lidocaine anesthesia reduces the incidence of postoperative airway events.

**Trial registration:**

ChiCTR, ChiCTR-IPR-17012347. Registered August 12, 2017.

## Background

Laryngeal mask airways (LMAs) have several advantages, including low stimulation, high airtightness, and ease of operation, in supraglottic airway management [1–3]. In addition, LMAs could reduce the incidence rates of perioperative adverse airway events in children and have been widely applied for general anesthesia in children [4]. However, laryngeal mask-related adverse airway events have also been reported, mainly in the recovery period after the operation, including upper airway obstruction, laryngeal spasm, hypoxemia, and even cardiac arrest [5]. Therefore, LMA management in the recovery period is critical, and close attention should be paid to the timing of LMA removal.

Currently, two opposing views regarding the timing of LMA removal after operation under general anesthesia have been reported, namely under deep anesthesia and in the awake state [6]. Many studies suggested LMAs be removed under deep anesthesia in children operated under general anesthesia; this could be associated with high airway responsiveness that could lead to adverse events, including cough, laryngeal spasm, and pharyngalgia (pain in the pharynx) when the anesthesia become lighter during the recovery period. Meanwhile, glossocoma (a retraction of the tongue causing airway obstruction) could occur with LMA removal under deep anesthesia, leading to upper airway obstruction and hypoxemia [7]. Others demonstrated that pediatric patients with an awake LMA removal show markedly more adverse events compared with the deep removal group [8]. Nevertheless, deep extubation is associated with a higher risk of obstruction (relieved by simple airway maneuvers), while awake extubation is associated with a higher risk of coughing and PACU complications [8, 9].

Previous reports have demonstrated that lidocaine improves LMA insertion and reduces the incidence rates of perioperative airway complications in children with upper respiratory infection [10, 11]. This may be explained by the fact that topical anesthesia could decrease LMA stimulation of the pharynx-larynx, consequently reducing adverse events, including cough and laryngeal spasm [12, 13].

Despite this wealth of knowledge, the timing of LMA removal after lidocaine anesthesia remains undefined. We hypothesized that applying lidocaine to the LMA cuff and removing the LMA in the awake state would reduce the incidence rates of airway complications, including glossocoma and upper airway obstruction. Therefore, the present randomized controlled trial aimed to assess the optimal timing for LMA removal and the effect of topical anesthesia with lidocaine on airway complications.

## Methods

### Study design and patients

In this randomized controlled trial, pediatric patients scheduled for squint correction surgery under general anesthesia in Tianjin Eye Hospital between September 1, 2017, and July 1, 2019, were included. The current study was registered on August 12, 2017 (No. ChiCTR-IPR-17012347), and approved by the Ethics Committee of Tianjin Eye Hospital (No. TJYYLL-2017-2). It strictly abided by the Declaration of Helsinki and CONSORT Standards. Written informed consent was obtained from the guardians of all the patients included in this study.

Inclusion criteria were: 1) age of 1–6 years; 2) scheduled selective squint correction surgery under general anesthesia; 3) ASA grade I-II; 4) informed consent from the parents or guardians.

Exclusion criteria were: 1) premature birth; 2) a history of upper respiratory infection within the last 2 weeks; 3) diseases associated with high airway responsiveness, including anatomically abnormal airway and bronchial asthma; 4) body weight < 9 kg or > 30 kg (if the weight is below or over the normal weight of the corresponding age range, the risk of surgical adverse airway events might be influenced [14, 15]); or 5) allergy to lidocaine, or history of arrhythmia, congenital heart disease, psychiatric disorders, or other disorder of psychological development. Patients who had an unsuccessful laryngeal mask insertion at the first attempt were withdrawn from the trial.

### Randomization and blinding

A random digital generator in the SPSS 21.0 software (IBM, Armonk, NY, USA) was adopted to divide the patients into four groups (1:1:1:1). After achieving the complete vacuum and plasticity of the LMA cuff in the LA and LD groups, the front and back sides of the cuff were evenly covered with lidocaine cream. In the NLA and NLD groups, water-soluble lubricant was applied to the cuff. The LMA was removed in the awake state (LA and NLA groups) or under deep anesthesia (LD and NLD groups).

The awake state was defined as the spontaneous opening of the eyes and mouth. Removal in the deep anesthesia groups was performed after operation completion and ventilation-associated recovery (respiration rate [RR] > 8 bpm and tidal volume ≥6 ml/kg). LMA removal was considered to be successful if it was accomplished without coughing, teeth clenching, gross purposeful movement, breath-holding, or laryngospasm, during or within 1 min after removal [16].

An attending anesthesiologist assessed the eligibility of patients and recorded their baseline data before surgery. Postoperative complications were assessed and recorded by an anesthesia nurse who did not participate in this study. All the operations were conducted by the same operation team.

The patients, guardians, and the data analyst were blinded. The anesthesiologist who conducted the anesthesia and removed the LMA and the anesthesia nurse who assessed the postoperative complications knew the grouping.

### Anesthetic management

A senior attending physician who did not participate in this study conducted anesthesia according to the information sealed in envelopes. Anesthesia in all patients was induced according to standard protocols, and no drugs were administered before the operation. Mask inhalation of 8% sevoflurane (oxygen flow of 5 L/min) was performed after the patient was transferred to the operating room, and the left lower extremity vein was accessed after the patient became unconscious. Then, 1 mg/kg of propofol and 0.1 μg/kg of sufentanil were intravenously administered. The LMA was inserted after the trapezius squeezing test showed no responsiveness. LMA insertion was conducted according to the Archie Brain standard method [17], and air inflation was performed via monitoring with a pressure meter to ensure an air pressure of 30 cmH_2_O. After optimal ventilation was confirmed, the LMA was fixed with tape. Inhalation of 3–4% sevoflurane was used for maintenance anesthesia (oxygen flow of 2 L/min), while the autonomous respiration of children was preserved. The end-tidal carbon dioxide partial pressure (P_ET_CO_2_) was maintained at < 55 mmHg, and pulse oxygen saturation at > 98%. Manually assisted ventilation was performed if necessary. For all patients, electrocardiographic (ECG) parameters, non-invasive blood pressure, pulse oxygen saturation (SPO_2_), P_ET_CO_2_, and bispectral index (BIS) were routinely monitored. After the patients completed the operation and met the criteria for transferring to the PACU (RR > 8 bpm, tidal volume > 6 mL/kg, and BIS < 60), the LD and NLD groups underwent LMA removal and were transferred to the PACU for further monitoring. The LA and NLA groups underwent LMA removal in the awake state in PACU. LMA removal in all patients was carried out by the same anesthesiologist who conducted the anesthesia. The pediatric patients with Aldrete score ≥9 were transferred to regular wards. Adverse airway events, the number of children with agitation (Pediatric Anesthesia Emergence Delirium [PAED] score > 12), and PACU stay were recorded [18]. The items monitored in the PACU were blood pressure (every 5 min), heart rate, electrocardiogram, pulse oxygen saturation, end-expiratory carbon dioxide partial pressure, and the score of agitation in the recovery period (using the PAED scale). Each nurse was responsible for only one patient at a time.

### Endpoints

The primary endpoint was a composite of irritating cough, laryngeal spasm (reflex spasm and contraction of throat muscles, which induces vocal fold adduction and partial or complete glottis closure, consequently leading to different degrees of dyspnea and even complete airway obstruction), SpO_2_ < 96%, and glossocoma (falling of the tongue under gravity, partially or completely blocking the airway when the patient is in supine position) in the recovery period in the PACU. The secondary endpoints included the incidence of pharyngalgia and hoarseness within 24 h after operation, duration of PACU stay, and incidence of agitation (consciousness disorder before being awake, characterized by physical and mental symptoms [19]) in the recovery period. Multiple airway events at the same time in the same patients were treated as one incident.

### Emergency treatment for adverse airway events

In patients with laryngeal spasm (reflex spasm and contraction of throat muscles, which induce vocal fold adduction and partial or complete glottis closure, consequently leading to different degrees of dyspnea and even complete airway obstruction), 1–2 mg/kg propofol (intravenous) was applied to enhance anesthesia, and high-pressure oxygen inhalation was provided. Succinylcholine (0.5–1 mg/kg, intravenous) and high-pressure oxygen inhalation could also be provided if necessary. Patients with glossocoma inducing upper respiratory obstruction were placed in the lateral position, and underjaw lifting or insertion of the oropharyngeal airway was performed. Any complications in the recovery period or within 24 h after the operation was considered an adverse airway event. No measures to reduce airway complications prophylactically were used, including IV lignocaine, IV dexamethasone, and pre-operative B agonists.

### Statistical analysis

Sample size estimation was performed according to a pilot study (unpublished), which showed overall incidence rates of adverse airway events of 10, 30, 40, and 30% in the LA, LD, NLA, and NLD groups, respectively. The sample size was calculated according to n $=\frac{2\overline{p}\overline{q}{\left({Z}_{\alpha }+{Z}_{\beta}\right)}^2}{{\left(p1-p2\right)}^2}$, where p_1_ is the incidence of the primary endpoint in the LA group (10%), p_2_ is the incidence of the primary endpoint in the NLD group (30%), p_mean_=(p_1_+p_2_)/2, q_mean_=1- p_mean_, and Z_α_ and Z_β_ are from the table of normal distribution (when α=0.05, Z_α_ is 1.96; when 1-β=0.9, Z_β_ is 1.28). Thus, the sample size was estimated as a two-group study, and the other two groups used the same sample size and were not adjusted for multiplicity. With a significance set at 0.05 and the power set at 90%, the calculated sample size was 84 in each group. Taking into account a lost-to-follow-up rate of about 10%, 93 patients were required in each group.

The SPSS 21.0 (IBM, Armonk, NY, USA) software was used for data analysis. Continuous variables were presented as mean±SD and compared by one-way analysis of variances (ANOVA) followed by post hoc least significant difference (LSD) tests. Categorical variables were presented as numbers and percentages and compared by the chi-square test or Fisher’s exact test. Z-test was used to compare the categorical variables between groups. *P*< 0.05 was considered statistically significant.

## Results

### Baseline patient characteristics

**Fig. 1:**
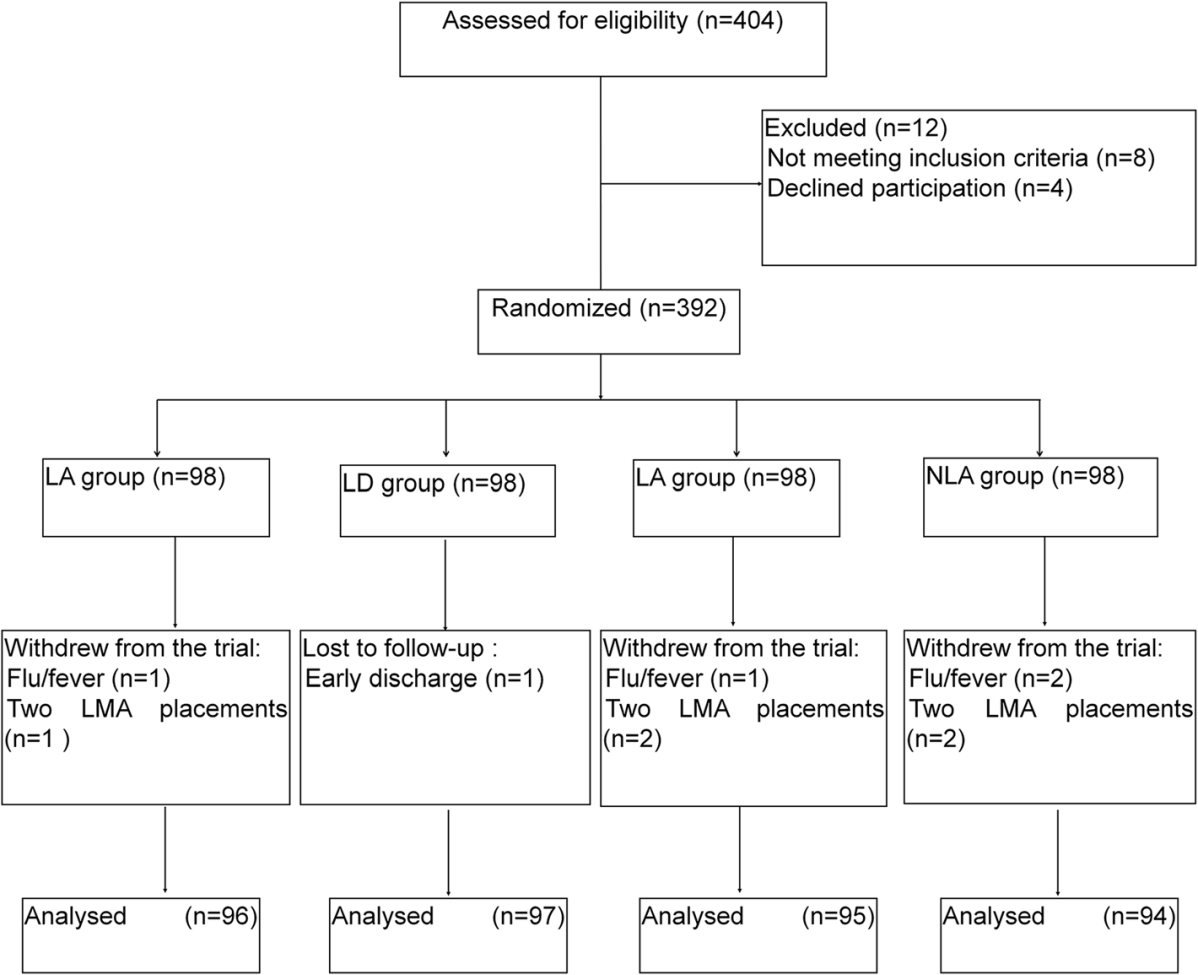
Study flowchart. LA group: lidocaine cream smeared to the cuff of the laryngeal mask airway (LMA) before insertion, with mask removal in the awake state. LD group: lidocaine application and LMA removal under deep anesthesia. NLA group: hydrosoluble lubricant application and LMA removal in the awake state. NLD group: hydrosoluble lubricant application and LMA removal in deep anesthesia

Of the 404 patients included, 12 were excluded, and the remaining 392 were randomized into the LA, LD, NLA, and NLD groups (*n*=98 per group). Finally, 96, 97, 95, and 94 patients completed this trial and were assessed in the LA, LD, NLA, and NLD groups, respectively. The study flowchart is shown in Fig. 1. All analyses were performed using the per-protocol set. There were no significant differences among the four groups in age, gender, BMI, ASA grade, and time of operation and anesthesia (all *P* > 0.05). None of the patients had a history of general anesthesia (Table 1). There were no differences among the four groups regarding the insertion conditions.

**Table 1:** General patient characteristics

Group	LA group (*n*=96)	LD group (*n*=97)	NLA group (*n*=95)	NLD (*n*=94)
Age (y)	3.9±1.1	3.9±1.1	3.9±1.2	3.8±1.2
Gender (M)	46 (47.9%)	48 (49.5%)	49 (51.6%)	47 (50.0%)
Weight (kg)	16.6±3.0	16.4±2.8	16.1±2.7	16.0±2.8
Operation time (min)	19.7±3.2	20.0±3.3	19.8±2.8	19.7±3.2
BMI (kg/m^2^)	15.8±1.1	15.7±1.3	15.4±1.0	15.5±1.3
ASA grade				
I/ II	96/0	97/0	95/0	94/0
Time of anesthesia (min)	26.3±3.1	26.5±3.4	26.2±2.8	26.1±3.1
History of general anesthesia	0	0	0	0

*M* male, *PACU* postanesthesia care unit, *BMI* body mass index, *ASA* American society of anesthesiologists

### Adverse events in the recovery period

The overall incidence of any adverse airway events was significantly lower in the LA group (9.4%) compared with the LD (23.7%), NLA (32.6%), and NLD (28.7%) groups (*P*=0.001). The incidence of cough in the NLA group (20.0%) was significantly higher in comparison with the LA (5.2%), LD (4.1%), and NLD (9.6%) groups (*P*=0.001). In addition, laryngeal spasm incidence was significantly higher in the NLA group (9.6%) compared with the LA group (0%) (*P*=0.001), and there was no significant difference among LA, LD (1%), and NLD (2.1%) groups. The incidence of low oxygen saturation (SpO_2_ < 96%) was significantly lower in the LA group (0%) compared with the LD (8.2%), NLA (13.7%), and NLD (9.6%) groups (*P*=0.005). Glossocoma incidence in the LA (0%) and NLA (0%) groups were significantly lower than those of the LD (19.6%) and NLD (20.2%) groups (*P*< 0.001). The incidence of agitation and duration of PACU stay showed no significant differences among the four groups (*P*=0.799, 0.980, respectively) (Table 2).

**Table 2:** Adverse airway events in the recovery period and within 24 h after operation

Group	LA group (*n*=96)	LD group (*n*=97)	NLA group (*n*=95)	NLD group (*n*=94)	*P* value
**Any adverse airway event**	9 (9.4%)	23 (23.7%)^a^	31 (32.6%)^a^	27 (28.7%)^a^	0.001
**Complications in recovery period**	5 (5.2%)	23 (23.7%)^a^	25 (26.3%)^a^	25 (26.6%)^a^	< 0.001
Cough	5 (5.2%)	4 (4.1%)	19 (20.0%)^ab^	9 (9.6%)	0.001
Laryngeal spasm	0 (0%)	1 (1.0%)	9 (9.5%)^ab^	2 (2.1%)^c^	0.001
SPO_2_< 96%	0 (0%)	8 (8.2%)^a^	13 (13.7%)^a^	9 (9.6%)^a^	0.005
Glossocoma	0 (0%)	19 (19.6%)^a^	0 (0%)^b^	19 (20.2%)^ac^	< 0.001
**Complications within 24 h after operation**					
Pharyngalgia	3 (3.1%)	1 (1.0%)	15 (15.8%)^ab^	3 (3.2%)^c^	< 0.001
Hoarseness	3 (3.1%)	2 (2.1%)	7 (7.4%)	2 (2.1%)	0.164
**Agitation in recovery period**	6 (6.3%)	4 (4.1%)	7 (7.4%)	5 (5.3%)	0.799
**Duration of PACU stay (min)**	15.1±2.7	15.1±2.4	15.2±2.5	15.1±1.8	0.980

*PACU* postanesthesia care unit. Any adverse airway event includes any complications in recovery period and within 24 h after operation

^a^*P*< 0.05 vs. LA group

^b^*P*< 0.05 vs. LD group

^c^*P*< 0.05 vs. NLA group; all adjusted using the LSD

### Adverse airway events at 24 h after operation

The incidence of postoperative pharyngalgia was significantly higher in the NLA group (15.8%) compared with the LA (3.1%), LD (1.0%), and NLD (3.2%) groups (*P*< 0.001). However, the incidence of postoperative hoarseness showed no significant differences among the four groups (*P*=0.164) (Table 2).

## Discussion

This randomized controlled study demonstrated that LMA removal in the awake state after topical lidocaine anesthesia reduces the incidence of airway events during the recovery period in pediatric patients.

LMAs in adults are generally removed in the conscious state; in contrast, it is generally suggested to remove them at the state of deep anesthesia in children. Park et al. [20] demonstrated that the incidence rates of SPO_2_ reduction and cough are higher after LMA removal in the conscious state compared with the deep anesthesia group, while airway obstruction incidence showed the opposite trend. After LMA removal under deep anesthesia, the oropharyngeal airway could be inserted, or the children could be placed in the lateral position to reduce glossocoma incidence. However, using the oropharyngeal airway could also introduce certain stimulations to the airway [21]. In addition, the lateral position could also damage nerves and blood vessels [22].

In this study, the overall incidence of adverse airway events was significantly reduced in the LA group compared with other groups, indicating the superiority of LMA removal with a combination of lidocaine application and awake state for removal. Airway complications such as coughing (related to awake state usually) showed a significant difference between NLA vs. other deep groups (LD, NLD) and also lidocaine application (LA). This corroborates previous external findings of a higher coughing rate with awake removals. It is not surprising that lidocaine application seems to have reduced these coughing episodes through pharyngeal anesthesia in the LA group, despite removal in the awake state. Nevertheless, in this study, the incidence of adverse airway events was higher than in previous studies [23, 24]. The difference may be due to the use of different age groups, different patient populations, different local practices, and different surgical procedures.

Changchien [25] and Bahk [26] have shown that topical anesthesia with lidocaine overtly improves the conditions for laryngeal mask insertion and reduces the dose of anesthetic agents. Indeed, topical anesthesia with lidocaine could reduce the conduction of stimulation from the laryngeal mask airway. As shown above, the application of lidocaine reduced the incidence rates of adverse events, including cough and laryngeal spasm, enabling children to well tolerate the LMA even in the state of light anesthesia or consciousness, and allowing patient placement in the supine position. The incidence of agitation during recovery and the time of PACU stay were not significantly different among the four groups. Applying lidocaine cream to the laryngeal mucosa could exert anesthetic effects, which consequently reduce the conduction of stimulation, and the muscle activities of the laryngopharynx after anesthesia become lighter [27, 28]. Therefore, the oppression and friction on the mucosa of the laryngopharynx by laryngeal mask was reduced, which consequently alleviated the tissue mucosal edema and discomfort [29, 30]. In the present study, the incidence of postoperative pharyngalgia was significantly lower in the LA group compared with the NLA group, and the laryngeal spasm also showed significantly lower incidence in the LA group than the NLA group. In addition, LMAs were preserved in the LA group during the recovery period, which prevented glossocoma and airway obstruction; consequently, low SPO_2_ incidence was decreased significantly.

The overall incidence of adverse airway events was significantly lower in the LA group compared with the remaining three groups, and major outcomes, including laryngeal spasm and SPO_2_ reduction, were improved as well. But there was no significant difference between LD and NLD groups; this indicates that lidocaine could not reduce the airway complications under deep anesthesia level. These findings suggested that laryngeal mask removal in the awake state under topical anesthesia with lidocaine has certain advantages in terms of safety performance. Meanwhile, the incidence rates of postoperative hoarseness and pharyngalgia did not increase significantly, suggesting that this method also has a certain degree of comfort.

There were several limitations to this study. First, it was a single-center study. Second, only one type of surgery was included, limiting the generalizability of the results. The duration of operation and anesthesia were relatively short in children undergoing squint correction surgery, and stimulation from pain is relatively low. Third, our choice of composite outcome was based on clinically relevant endpoints observed during deep and awake extubation. However, the use of composite outcomes can make individual differences less obvious and make some groups appear similar. For example, the LD group had a lower rate of laryngeal spasm and a higher rate of glossocoma than the NLA group; however, when discussing the composite outcome, they were similar. We recognize the potential for a higher type 1 error rate due to multiple outcomes and testing. We attempted to adjust for this by using adjustment methods such as LSD. Fourth, all complications were treated in the same way irrespective of the different phases of anesthesia. Fifth, the patients in the awake group had their LMA removed in the PACU instead of the operating room, and the environmental conditions are different and could affect the outcomes. Sixth, no screening for allergies was done, and this could bias the results regarding airway stimulation. Finally, the assessor was unblinded. Therefore, further studies are required to verify the present findings and expand them to other operation types. This study investigated the influence of two interventions (timing of LMA removal and use of lidocaine or not) on the airway complications during the recovery period of children under anesthesia. The results may provide some guidance for clinical decision-making.

## Conclusions

LMA removal under topical anesthesia with lidocaine in the awake state could reduce the incidence rates of airway events in the recovery period in pediatric patients undergoing general anesthesia.

## Data Availability

The datasets used and/or analyzed during the current study are available from the corresponding author on reasonable request.

## Abbreviations

LMAs: Laryngeal mask airways
PETCO2: End-tidal carbon dioxide partial pressure
ECG: Electrocardiographic
SPO2: Pulse oxygen saturation
BIS: Bispectral index
